# Low-Resource Time-to-Digital Converters for Field Programmable Gate Arrays: A Review

**DOI:** 10.3390/s24175512

**Published:** 2024-08-26

**Authors:** Diego Real, David Calvo

**Affiliations:** IFIC—Instituto de Física Corpuscular, CSIC—Universitat de València, c/Catedrático José Beltrán, 2, 46980 Paterna, Spain; dacaldia@ific.uv.es

**Keywords:** time-to-digital converter, field-programmable gate array, low-resource time-to-digital converters

## Abstract

A fundamental aspect in the evolution of Time-to-Digital Converters (TDCs) implemented within Field-Programmable Gate Arrays (FPGAs), given the increasing demand for detection channels, is the optimization of resource utilization. This study reviews the principal methodologies employed for implementing low-resource TDCs in FPGAs. It outlines the foundational architectures and interpolation techniques utilized to bolster TDC performances without unduly burdening resource consumption. Low-resource Tapped Delay Line, Vernier Ring Oscillator, and Multi-Phase Shift Counter TDCs, including the use of SerDes, are reviewed. Additionally, novel low-resource architectures are scrutinized, including Counter Gray Oscillator TDCs and interpolation expansions using Process–Voltage–Temperature stable IODELAYs. Furthermore, the advantages and limitations of each approach are critically assessed, with particular emphasis on resolution, precision, non-linearities, and especially resource utilization. A comprehensive summary table encapsulating existing works on low-resource TDCs is provided, offering a comprehensive overview of the advancements in the field.

## 1. Introduction

Time-to-Digital Converters (TDCs), which convert a pulse time duration into a numeric value, are used in various applications where an accurate measurement of time is needed, such as time-based signal processing [1], ASIC testing [2], detecting Trojan infections in ASIC [3], nuclear medicine imaging with Positron Emission Tomography [4,5,6,7], measuring the laser flight time in laser ranging [8], fluorescence spectroscopy [9], ultrawideband radio frequency localization [10,11], Laser Imaging Detection and Ranging [12], or experiments in high-energy physics [13,14,15,16], including neutrino telescopes [17,18,19] and accelerator instrumentation [20,21,22].

TDCs can be implemented in both ASICs and FPGAs. ASICs can provide better performances, especially when there are requirements for high resolution. Nevertheless, FPGAs are less expensive and complex than ASICs; they provide a faster development time and the flexibility to adapt the logic to the operation conditions, reducing the development costs and the time-to-market time [23,24,25,26,27]. Furthermore, the remaining FPGA resources can be utilized to process TDC data and interface with the rest of the readout system, which results in higher reliability [28,29,30].

There is an increasing demand for a higher number of TDC channels implemented in FPGAs, which requires low-resource and low-power TDCs. FPGAs are devices designed to operate synchronously; therefore, the primary and simplest method for implementing TDCs consists of a simple counter. The resolution of this basic implementation can be extended using various methods, allowing for resolutions significantly finer than the period of the counter clock. To extend the counter’s resolution, Nutt architecture (also called interpolation archictecture) [31] is employed in FPGAs. The counter keeps track of the clock cycles, while an interpolator computes the fractional part within the clock cycle for both the start and stop segments.

The most used low-resource interpolation methods is the Multi-Phase Shift Clock (MPSC) together with the recently introduced Gray Code Oscillator (GCO), while there have also been advances to reduce the number of resources used in Tapped Delay Line (TDL) and Vernier Ring Oscillator (VRO) TDCs. A special type of TDC is based on a two-step interpolation, each of the two using different interpolation techniques. Noise-Shaping (NS) TDCs have recently been implemented fully in FPGAs, although the use of resources is still considerable. The advances in low-resource TDCs, with their advantages and drawbacks, are reviewed in this work. A summarizing table shows the existing works on low-resource TDCs reviewed in this work. The counter architecture, the simplest method to implement TDCs in FPGAs, is presented in Section 3. The main parameters describing TDCs are detailed in Section 2, while Section 4 is dedicated to TDL-TDCs, Section 5 discusses VRO-TDCs, NS-TDCs are analyzed in Section 6, GCO-TDCs are presented in Section 7, MPSC-TDCs are reviewed in Section 8, and Section 9 is dedicated to two-stage interpolation TDCs. Finally, a discussion takes place in Section 10, while the conclusions are presented in Section 11.

## 2. TDC Main Parameters

The performance and characteristics of a TDC are defined by several key parameters. The most important of them include the following:

- Resolution refers to the smallest detectable time interval or the smallest distinguishable step-in-time measurement that the TDC can achieve. The smallest resolvable time interval is represented by the Least Significant Bit (LSB), which establishes the resolution of the TDC.
- Dead-time refers to the minimum time interval required between two consecutive events or pulses for accurate detection and measurement. It is the period during which the TDC is temporarily unable to respond to incoming signals due to internal processing, resetting, or recovery time. Dead-time is an inherent characteristic of TDCs and is primarily caused by the time required for data acquisition, signal conditioning, digitization, and internal circuitry operations.
- Precision refers to the repeatability and consistency of time measurements performed by the TDC. It is directly affected by the non-linearities of the TDC.
- Differential non-linearities (DNLs) can be defined as the deviation of a single quantization step from the ideal LSB. They quantify the step size variation between consecutive output codes. DNL is typically expressed as the difference between the measured step size and the ideal step size (in LSB). The DNL is evaluated by comparing the number of pulses per LSB bin ($n_{i}$) with the mean value ($\bar{n}$):
  (1) $${\mathrm{DNL}}_{i}=\frac{n_{i}-\bar{n}}{\bar{n}}$$
- Integral non-linearities (INLs) describe the greatest deviation in the transfer function of a TDC from the ideal linear relationship. The INL value provides information about the linearity and precision of the TDC’s measurements. It can be determined by performing the following calculation [18]:
  (2) $$\mathrm{INL}=\frac{\bar{T}-T_{\mathrm{in}}}{t_{\mathrm{bin}}}$$
  where $T_{\mathrm{in}}$ represents the input pulse width, $\bar{T}$ denotes the average of the pulse width measurements, and $t_{\mathrm{bin}}$ indicates the bin size.
- Range refers to the time intervals that the TDC is capable of accurately measuring. It represents the minimum and maximum values that the TDC can handle within its specified operating conditions.
- Clock Frequency refers to the frequency at which the TDC’s internal clock operates, influencing the resolution and measurement capabilities.
- Calibration parameters: some TDCs, in order to compensate for non-linearities and enhance accuracy, require a calibration process. Usually, the calibration system increases the use of resources.
- Resource occupancy concerns the utilization of FPGA resources, such as lookup tables (LUTs), flip-flops (FFs), memory blocks, interconnects, or other FPGA hardware, such as SerDes or IOs delays, to implement the TDC functionality. The present review is dedicated to TDCs which are implemented with low resource use.

## 3. The Basics: The Counter and the Interpolator

The simplest method to implement a TDC in an FPGA is the counter method, where the time interval is measured by counting the number of clock cycles that occur between two events. The resolution of the TDC is determined by the clock frequency. In FPGAs, the frequency is limited to a few hundred megahertz [32,33], which, in turn, limits the performances of the TDCs. One of the main advantages of this method is that non-linearities are minimal and dependent on the clock jitter. Another advantage is that the TDC’s scale can be extended as needed, simply by increasing the counter’s range.

**Figure 1:**
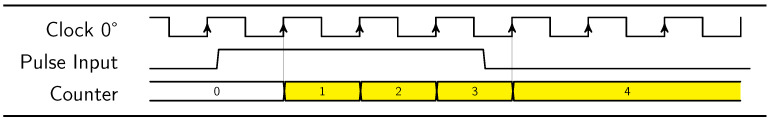
Timeline of a TDC based on a counter. The resolution is limited by the period of the clock.

The range of the TDC can be calculated as follows:

(3) $$\mathrm{Range}=\left(2^{n}-1\right)\times T_{clk}$$

where range is the maximum measurable time interval, *n* represents the number of bits in the counter, and $T_{clk}$ is the period of the clock signal. By increasing the number of bits in the counter, the total countable range of the TDC is also increased. For each additional bit added, the TDC’s measurable range is effectively doubled. As shown in Figure 1, the start and end of the pulse input are not properly measured. To enhance the TDC’s resolution, the interpolation method (also called Nutt’smethod) is employed. This approach delivers a greater resolution. The counter provides a coarse count, while the time intervals before the first counting cycle and after the last one are interpolated with a finer resolution method. The counter keeps track of the clock cycles, while the interpolator computes the fractional part within the clock cycle in both the start and stop signals with respect to the clock edge. In the realm of Field-Programmable Gate Arrays (FPGAs), two primary interpolation methods are employed to implement low-resource TDCs: MPSC and GCO, while other interpolation methods such as TDL and VRO have achieved considerable reductions in the use of resources. Each of these methods will be thoroughly detailed in the subsequent sections.

## 4. Tapped Delay Line TDCs

**Figure 2:**
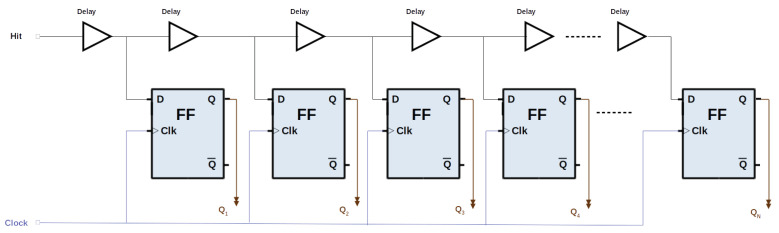
Basic TDL architecture. The delay line goes through a line of FFs which are clocked with the same clock.

The TLD technique relies on a logic buffer delay cell, where the start signal propagates and the stop signal latches the state of the delay line in a register (see Figure 2). The resolution is determined mainly by the basic delay duration, while the precision is determined by the uniformity of the delay tap. When the rising edge of the master clock signal detects the start or end of a pulse at the input of the TDC, all the FFs in the TDL are frozen and read, enabling a more precise measurement of the time interval [34]. Different alternatives have been explored to implement the TDL in FPGAs. The FPGA routing resources can be used to create the basic delay and can also be used to implement vernier TDCs [29]. Other elements to implement the TDL are FFs [35] and LUTs [36]. DSP blocks within FPGAs have also been used to implement TDLs [37], while the most widely adopted approach for implementing TDL TDCs is the use of the arithmetic carry propagation primitive [38].

Recent implementations of TDL-TDC using carry lines have achieved a <10 picosecond resolution [39,40]. However, it is important to note that the arithmetic carry, when implemented in an FPGA, does not exhibit linear delay behavior. As a result, TDL architectures relying on arithmetic carry methods can suffer from high levels of differential and integral non-linearity errors (DNLs and INLs). These errors manifest as some of the tapped delays and do not follow the programmed order, a phenomenon known as “bubble error”. Bubble error directly impacts the precision of TDCs and introduces non-linearities into the measurements. Additional variations can occur due to periodic patterns arising from the Configurable Logic Block (CLB) structure and the clock distribution tree boundaries within the FPGA. It is essential that the cumulative delay in the chain exceeds the period of the coarse counter clock to ensure accurate time measurements. Implementing TDL-TDCs with carry chains leads to higher system complexity and the intensive utilization of FPGA resources. Achieving uniform delays often requires manual routing, which adds additional complexity to the development process. To enhance the intrinsic resolution of the TDL beyond what is limited by the granularity of the basic delay, TDL-TDCs have been used in parallel [41]. By using 125 TDL-TDCs of 323 ps in parallel, a resolution of just 5.8 ps has been achieved [42]. However, the use of several TDCs in parallel considerably increases the use of resources.

The recirculation of the pulse through the TDC is another method [43]. This approach allows for significant enhancements in performance by combining results from multiple measurements. The Wave Union (WU) methods represent an evolution of this concept [44]. In these two methods, the tapped delay is subdivided into smaller segments, improving the resolution through multiple measurements. The WU A method boosted the resolution by 37%, while the WU B method achieved a 75% increase. However, these gains in resolution come at the cost of increased dead time, heightened complexity, and greater resource usage. Furthermore, in FPGAs manufactured with 28 nm or lower, the WU method poses greater challenges [45]. Nonetheless, architectural enhancements have led to subpicosecond resolution achievements [46,47]. Notably, implementing the WU method with DSP blocks has resulted in superior performance [48].

TDL-TDCs, in any of their variations, are intended for high resolution and are not Power, Voltage, and Temperature (PVT) compensated (one exception is the TDL created with IODELAY primitives); they are highly non-linear, which requires calibration, and are highly intensive in the utilization of FPGA resources. TDL-TDCs consume high resources when compared with other architectures with higher efficiencies [39]; nevertheless, recently, some advances have been made in the area of low resources and Parsa et al. developed a TDL-TDC with reduced resource consumption. Although resolution and precision are high (<30 ps), both the non-linearities and the number of resources are still considerable, especially the consumption of BRAM blocks [49,50], which is as high as 90 kB per TDC channel. The implementation has been carried out in an Artix-7 FPGA, where DNLs of 2.13 LSBs have been achieved in the first implementation and 1.18 LSBs in the optimized version (INLs of 3.97 and 2.75 LSBs respectively), with consumptions of 216 LUTs and 678 FFs in both implementations.

### Expanding Precision and Resolution beyond the Delay Cell

To enhance the intrinsic resolution of the TDL beyond what is limited by the granularity of the basic delay, TDL-TDCs can be used in parallel [41]. By using 125 TDL-TDCs in parallel at 323 ps, a resolution of just 5.8 ps has been achieved [42]. A parallel multichain cross segmentation method without multitime measurements has also been proposed, achieving a 1.3 ps resolution and a 4.6 ps single-shot precision [51]. The recirculation of the pulse through the TDC is another method [43]. This approach allows for significant enhancements in performance by combining results from multiple measurements. The Wave Union (WU) methods represent an evolution of this concept [44]. In these two methods, the tapped delay is subdivided into smaller segments, improving resolution through multiple measurements. The WU A method boosted the resolution by 37%, while the WU B method achieved a 75% increase. However, these gains in resolution come at the cost of increased dead-time, heightened complexity, and greater resource usage. Furthermore, in FPGAs manufactured with 28 nm or lower, the WU method poses greater challenges [45]. Nonetheless, architectural enhancements have led to subpicosecond resolution achievements [46,47]. Notably, implementing the WU method with DSP blocks has resulted in a superior performance [48]. The most recent breakthrough involves a refinement in the architecture, wherein solely the initial multiplexer is employed to generate the pulse train for Wave Union upon receipt of the start signal. This modification aims to significantly reduce the necessary computational processing, optimizing overall efficiency [52].

## 5. Vernier Ring Oscillator TDCs

**Figure 3:**
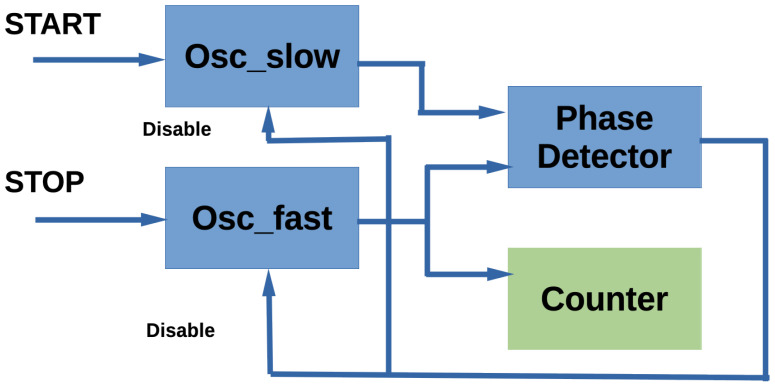
Vernier Ring Oscillator TDC basic scheme. The resolution is determined by the frequency difference between the two oscillators.

The Vernier TDC is based on a reference delay line and a delayed signal path, both with slightly different propagation delays [53,54]. By comparing the delayed signal with the reference, the TDC determines the time difference between the events through an interpolation-like process, providing enhanced resolution beyond the clock resolution. A subtype of vernier TDCs, the Vernier Ring Oscillator (VRO)-TDC can be implemented in a very efficient manner, reducing the number of resources used [55] (see the basic scheme in Figure 3). The VRO-TDC is based on two external oscillators of different frequencies [28]. The oscillators are controlled with the start and stop signals of the pulse, the duration of which has to be measured. The highest frequency oscillator (with $T_{f}$ period) is initialized by the start signal and the lower frequency oscillator (with $T_{s}$ period) is initialized by the stop signal. After N cycles, both oscillators will be aligned and $T_{in}$ will be the time difference between the start and stop signals: $T_{in}=N\left(T_{s}-T_{f}\right)$. The TDC accuracy is determined by the difference between the periods of the two oscillators. The dead-time is sensibly high as the TDC has to wait until both clocks are aligned. Cui et al. have implemented low-resource VRO-TDCs with similar resolutions (<30 ps) to those achieved by Parsa et al. with TDL-TDCs, with a higher number of registers but with no use of BRAM blocks and better non-linearities [56]. A higher linearity has been achieved with this type of TDC but with a higher consumption of resources [57] (172 LUTs and 986 FFs versus 104 LUTs and 319 FFs of the first implementation).

## 6. Noise-Shaping TDCs

ΣΔ TDCs have been extensively used to measure the time delay between two repetitive digital signals (or clocks) with high precision. The primary drawback of this technique is its inability to measure a single-event input. An additional limitation arises from the requirement for certain analog components, such as an integrator and a comparator, which complicates the full implementation in FPGAs [58]. Recently, advancements have been achieved in overcoming these challenges. A sophisticated type of ΣΔ TDC, specifically a high-order continuous-time multi-stage noise shaping (MASH) 16 ΣΔ TDC, has been successfully integrated entirely within an FPGA. This was achieved by utilizing a Gated Switched-Ring Oscillator (GSRO) [59]. This integration represents a significant milestone in the field. Despite achieving a remarkably high resolution of 0.18 ps, the implementation by Khaki et al. still involves substantial resource consumption. While only 311 registers are used, the requirement for five phase-locked loops (PLLs) and, notably, 2 Mb of memory, precludes this type of TDC from being classified as low-resource at this stage. The progress in this area is promising, but further optimization is necessary to reduce resource utilization and enhance the feasibility of widespread low-resource FPGA implementation.

## 7. Gray Code Oscillator TDCs

Wu et al. proposed an innovative TDC architecture base in a free-running gray code oscillator [22]. Instead of using a synchronous counter, a combinational counter is implemented, running much faster, and thus increasing the resolution, reducing the resource utilization and power consumption and providing high scalability and portability. The gray code needs to be used, as being combinational and not having the delay time controlled, only one-bit transition propagates back to the input, so glitches are not generated, which would appear if another type of code were used. The implementation of the gray counter consumes very low resources, which are compensated by the necessary logic to improve the linearity via calibration. The drawbacks of this architecture are that the TDC bins have high non-linearities and that, as is the case with the previous solutions, they are not PVT-compensated. Upgrades in the architecture have been achieved to improve the non-lineareties [60] and the resolution [12]. The latest improvement in this architecture, achieved by Wang et al., has reached a resolution and precision below 40 picoseconds; nevertheless, the use of resources is still high, requiring 437 LUTs, 368 FFs, and 54 kB of RAM per channel in a Virtex-7 FPGA, San Jose, CA, USA [61].

## 8. Multi-Phase Shift Clock TDCs

**Figure 4:**
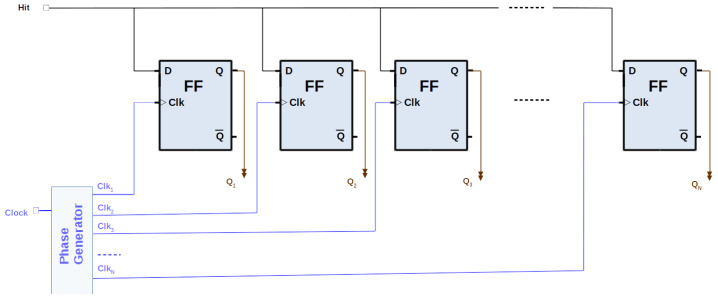
Oversampling scheme: the basis of the MPSC-TDC. The resolution is increased by the number of phases.

**Figure 5:**
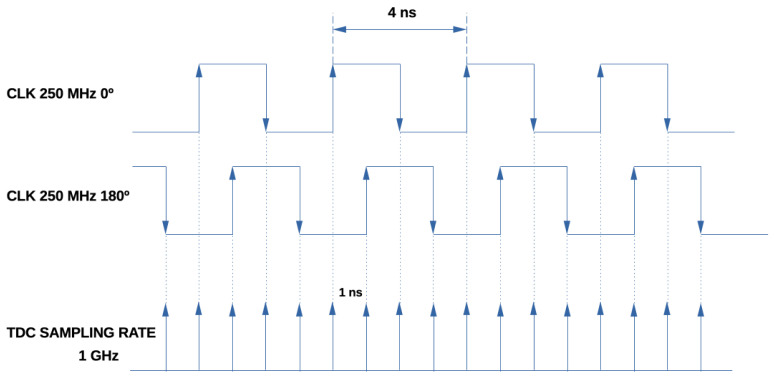
Oversampling timeline. In this case, the basic counter resolution is expanded by four by using the rising and falling edges of two clock signals shifted 180°.

The MPSC method utilizes several clocks with the same frequency but different phases, oversampling the TDC input (see Figure 4). Each clock monitors the TDC line, latching the status at the rising edge of the clock and thereby multiplying the resolution achievable by a single clock (see Figure 5). It is resource-efficient, enabling hundreds of channels in one FPGA. The resolution is increased by the number of phases used. The MPSC-TDCs usually have a reduced DNL, as the feature is intrinsic to the architecture and related to the jitter of the clocks. The main drawbacks are the limited number of clock lines the FPGA can support, the fan-out, and, in particular, the skew on the lines from the input signal to the FFs that could be much bigger than the requested resolution. With manual placement and routing, it is possible to improve the skew of the FFs below the TDC resolution, but these methods add complexity to the implementation. If the time path between two consecutive phases is bigger than the LSB, the signal of that specific phase will be sampled at the following clock edge; then, the final timestamp will not match with the real measure. This limits the resolution that can be achieved with MPSC-TDCs.

One of the first implementations of this type of TDC was carried out by Fries et al. [62], who achieved a resolution of 1400 ps and a DNL of 0.5 LSB. A higher resolution, 138 ps, was obtained by Yonggang et al. [63], who used eight clock phases. Balla et al. developed an MPSC-TDC [16] with four phases to obtain an LSB of 625 ps and a precision of 255 ps. The total numbers of LUTs and registers were 68 and 274, respectively. The number of registers included a buffer for 32 measurements (40 bytes in total), although the TDC did not compute the arrival time, which would have increased the consumption of resources. The linearity of the TDC was high, with a DNL of 0.05 LSBs and INL of 0.05 LSBs. A TDC with 16 phases was implemented by Büchele et al. [64], obtaining a resolution of 160 ps and a precision of 64 ps. The number of LUTs consumed by the TDC channel in a Virtex-5 FPGA was 125, while the number of registers was 198. Suwada et al. [65] and Li et al. [66] have implemented 1000 ps resolution TDCs in Spartan-6 FPGAs. Sano et al. have implemented TDCs with a resolution of 280 ps in a Kintex-7 FPGA [67] and of 780 ps in an IGLOO-2 FPGA [68], in the latter case for higher tolerance to irradiation. Jia et al. have achieved a resolution of 138 ps in an Artix-7 FPGA by using a clock of 300 MHz and 24 different phases [69].

### 8.1. SerDes TDC

A variant of the MPSC-TDC is based on the SerDes, short for Serializer/Deserializer. The SerDes plays a crucial role within an FPGA by converting a high-speed serial data stream into parallel data for reception purposes. The SerDes block can be employed to implement the oversampling method, making it possible to create four sampling phases with a single SerDes. Since the SerDes are integrated hardware components connected to the I/O, they occupy minimal FPGA resources. By multiplexing several SerDes, it is possible to increase the resolution, as it is equivalent to increasing the number of phases.

#### 8.1.1. One-SerDes TDCs

Several TDCs have been implemented with one SerDes. A TDC with a resolution of 1.2 ns using a Stratix FPGA [70] has been created by Fries et al. The jitter in the clock driven by the PLL is specified to be under 200 ps, resulting in a DNL of less than 200 ps. A TDC with a 1 ns resolution [18] using a Kintex-7 FPGA was implemented by Calvo et al., with DNLs of 0.03 LSBs and INLs of 0.12 LSBs. A higher resolution, 100 ps, has been achieved by Kong et al. [71]. The precision achieved is 169 ps, and it has been obtained in a Kintex-7 by using the SerDes to read 10 GBPS signals, which generates a 32-bit array that is needed to identify the rise and fall edges inside. The number of transceivers of 10 GBPS is limited in the FPGAs; therefore, the number of TDC channels that can be implemented using this type of TDC is also limited and cannot be used in all the inputs of the FPGA, as is the case with the usual SerDes.

#### 8.1.2. Two-SerDes TDCs

A TDC with a resolution of 156 ps has been proposed by Xiang et al. [72]. The TDC was implemented with two complementary SerDes working at 800 MHz and with eight different phases separated 45°. The resolution obtained was 56 ps, the INL was 1 LSB, and the DNL was 0.36 LSBs. The resource usage was only 109 LUTs and 238 registers, in addition to the two SerDes, implemented in an Artix-7 FPGA. Bai et al. [73], by using two SerDes and one IODELAY primitive, have achieved a resolution of 803 ps and a precision of 229 ps using only 30 LUTs and 42 FFs. The DNL was also very low (0.05 LSBs).

#### 8.1.3. Four-SerDes TDCs

Four SerDes acquiring in parallel, with the TDC input delayed one quarter of the SerDes acquisition period, have been used to increase the resolution even more, which would be the equivalent of an MPSC-TDC with 16 phases. The TDC developed by Arpin et al. [7] achieved a resolution of 321 ps in a Virtex-4, with a precision of 56 ps. The DNL was 0.3 LSBs and the INL was 1 LSB. A similar resolution was obtained by Imrek et al., who also used a Virtex-4. In this case, the DNLs were slightly higher (0.6 LSB). The latest TDC implementation based on this architecture has a resolution of 100 ps. This TDC has been implemented in a Kintex-7 FPGA using the SerDes working with a 625 MHz clock, which gives a total resolution of 42 ps [74]. In order to achieve a higher resolution, four SerDes TDCs are used in parallel, with the three last TDCs acquiring the signal delayed 100, 200, and 300 ps. The delays between the TDC subsections were achieved utilizing the IODELAY primitive, which allows a granularity in the delays of 39 ps. The main problem related to the use of the IODELAYs is that the IODELAY granular delay reported by Xilinx is only an average, and there exists a significant difference between the delay bins. This leads to high non-linearities, which are not reported in this work. The granularity of the IODELAY decreases in the new families, where it is expected that this architecture will increase the linearity. In any case, the main drawback of this architecture is the number of SerDes available in an FPGA, which is one per IO pin, so the use of four SerDes limits the number of TDCs that can be implemented to one quarter of the FPGA inputs.

## 9. Two-Stage Interpolation TDCs

The use of two-stage interpolation in TDCs has been studied as it can increase the resolution while the resources are kept low. In the first stage, the TDC performs a coarse measurement of the time interval. This is usually done with a lower resolution and is faster. The coarse measurement provides a rough estimate of the time interval, which is then used to narrow down the range for the second stage. In the second stage, the TDC performs a fine measurement of the time interval within the range provided by the coarse measurement. This stage uses a higher resolution method to achieve greater accuracy. Techniques such as delay line or Vernier methods are often employed here. By dividing the measurement into two stages, the TDC can achieve very high resolution and precision. The coarse stage provides a broad measurement, while the fine stage refines this measurement to a much higher degree of accuracy. The coarse stage typically consumes less power because it operates at a lower resolution and can perform the initial measurement quickly. The fine stage, while more power-intensive, only needs to operate for a shorter period within a reduced range. The two-stage approach allows the TDC to quickly get a rough measurement before spending more time and resources on refining the measurement. This can improve the overall speed of the conversion process. On the other hand, implementing a two-stage TDC is more complex than a single-stage TDC. It requires careful calibration and synchronization between the two stages to ensure accurate measurements. Dong et al. [75] have designed a TDC based on SerDes that increases the resolution with an additional TDL. The resolution obtained is 78.13 ps and the precision is 35 ps, while, in addition to the SerDes, 199 LUTs and 347 registers are used. Instead, the DNL and INL are somewhat higher at 0.8 and 0.94 LSBs, respectively.Real et al. [76] have implemented an MPSC-TDC with four phases that has been expanded with a one-tap delay by using the IODELAYs of Artix-7. The resolution achieved is 416 ps and the precision is 186 ps. The non-linearities are low (DNL 0.2 and INL 0.15 LSB) while the use of resources is quite contained: 102 LUTs and 115 registers. A significant advantage of this architecture, in addition to the low resources, is that the TDC is PVT-controlled. An additional architecture based on two-stage interpolation is the one developed by Wang et al. [77], which uses as first interpolator a GCO-TDC and expands it with a TDL of CARRY4s. The resolution achieved in a Virtex-7 is 10 ps, with a precision of 19.81 ps. The DNLs and INLs are high at 2.85 and 13.61 LSBs, respectively. The resource consumption is 293 LUTs and 385 registers. A drawback of this implementation is that is not PVT-compensated, as happens with both the TDL based on CARRY4 and the GCO TDCs.

## 10. Discussion

The TDL-TDCs provided the highest precision but their main drawback is that resource consumption is not as reduced as with other architectures. This can be explained by the number of registers needed to latch the TDL. They also present high non-linearities, which requires calibration of the TDC bins. Additionally, the delays are not PVT-compensated. An exception to this is if the IODELAY is used; however, in this case, the number of taps is limited by the IOs of the FPGA. VRO-TDCs have similar characteristics to TDL-TDCs, with a somewhat higher use of resources but with lower non-linearities. NS TDCs have been recently implemented completely, in particular a ΣΔ MASH TDC; however, the consumption of resources should still be improved. The GCO TDCs are a novel architecture that provide high resolution and precision, as well as low non-linearities with moderate use of resources. MPSC-TDCs are the most resource-effective technology, with generally low non-linearities and moderate resolution and precision. When the SerDes of the FPGA are used, the resource consumption can be reduced even more. Some combinations of interpolators achieve high resolutions, while the use of resources is kept at a minimum. Especially promising is the use of IODELAYs as the second interpolator, as this allows an increase of the resolution by tapped delays which are PVT-compensated. This feature is expected to improve performances in the latest version of FPGAs with the same consumption of resources, as both the granularity of the delay taps and the operation frequency are greater. The integration of the IODELAY architecture with an MPSC-TDC implemented using SerDes holds promising prospects for high efficiency, primarily due to the utilization of specific hardware resources rather than FPGA logic. This approach capitalizes on the strengths of both technologies to achieve an optimal performance while conserving resources. This study has exhaustively examined the principal methods for implementing TDCs in FPGAs, elucidating their respective advantages and drawbacks, as summarized in Table 1. These findings have been succinctly summarized in Table 2, providing a convenient reference for comparing and contrasting the various approaches.

## 11. Conclusions

There is a growing need for TDC channels implemented in FPGAs. This work has reviewed the heterogeneous methods to implement low-resource TDCs, while critically evaluating the benefits and drawbacks of each approach, focusing on factors such as resolution, accuracy, non-linearities, and, particularly, resource utilization. By critically evaluating each approach, this research furnishes a comprehensive overview of the existing studies on low-resource TDCs. Through this, it offers valuable insights into the progression of low-resource TDCs, thereby offering a comprehensive overview of progress in the field.

## Figures and Tables

**Table 1:** State-of-the-art TDC implementation comparison. For each of the works, the resolution, precision, non-linearities, and resource consumption are presented. The works are organized by the architecture used in the implementation of the TDC. Note than the PLLs of the MPSC TDCs are not included.

Work	Res. (ps)	Pre. (ps)	DNL/INL(LSB)	LUTs	FFs	Primitive	BRAM (kB)	FPGA
			**NS**					
**Kha-21 [59]**	0.18	NR	NR/NR	NR	311	5 PLL	2000	Stratix-IV
			**TDL**					
**Choi-21 [39]**	4.88	8.03	0.51/0.51	2962	4157	0	0	Artix-7
**Pars-21 [49]**	22.2	26.04	2.13/3.97	216	678	0	90	Artix-7
**Pars-22 [50]**	22.1	22.35	1.18/2.75	216	678	0	90	Artix-7
**Lusa-23 [78]**	156.25	93	0.23/0.26	295	446	0	0	Artix-7
**Lusa-23 [78]**	312.5	93	0.26/0.2	238	431	0	0	Artix-7
**Lusa-23 [78]**	625	255.5	0.045/0.045	212	336	0	0	Artix-7
			**VRO**					
**Cui-17 [56]**	[23, 37]	[32, 39]	0.8/1.7	104	319	0	0	Virtex-6
**Cui-20 [57]**	24.5	28	0.45/0.85	172	986	0	0	Virtex-6
			**GCO**					
**Wu-19 [22]**	256	160	1.25/NR	NR	NR	0	NR	Kintex-7
**Mach-20 [60]**	380.9	290	0.76/0.71	NR	NR	0	NR	Virtex-7
**Arau-21 [12]**	69	54.99	1.76/1.5	NR	NR	0	NR	Ultrascale+
**Wang-23 [61]**	20.97	17.11	0.087/0.224	455	368	0	54	Ultrascale+
**Wang-23 [61]**	36.01	27.37	0.102/0.262	453	367	0	54	Ultrascale
**Wang-23 [61]**	34.84	32.33	0.078/0.203	437	368	0	54	Virtex-7
**Wang-23 [61]**	256.41	N/R	0.65/3.1	380	333	0	54	Virtex-7
			**MPSC**					
**Frie-02 [62]**	1400	750	0.4/NR	NR	NR	0	0	Virtex-5
**Buch-12 [64]**	160	64	0.4/NR	125	198	0	0	Virtex-5
**Wang-13 [63]**	138	NR	0.29/NR	NR	NR	0	0	Virtex-5
**Ball-14 [16]**	625	255	0.05/0.05	68	274	0	0	Virtex-5
**Suwa-15 [65]**	1000	500	0.52/0.39	NR	NR	0	0	Spartan-6
**Sano-16 [67]**	280	NR	0.28/0.3	NR	NR	0	0	Kintex-7
**Sano-17 [68]**	780	350	0.53/2.8	NR	NR	0	0	IGLOO-2
**Li-17 [66]**	1000	430	NR/NR	NR	NR	0	0	Spartan-6
**Jia-18 [69]**	138	73.6	NR/NR	NR	NR	0	0	Artix-7
**Szplet-19 [79]**	43	36	1.91/NR	NR	NR	0	0	Kintex-7
			**MPSC SerDes**					
**Bogd-05 [70]**	1200	1200	0.17/NR	NR	NR	1 SER	0	Stratix
**Calv-21 [18]**	1000	NR	0.03/0.12	NR	NR	1 SER	0	Kintex-7
**Kong-23 [71]**	100	169	NR/NR	NR	NR	1 SER	0	Kintex-7
**Xian-14 [72]**	156	56	0.32/1	109	238	2 SER	0	Artix-7
**Bai-17 [73]**	803	229	0.05/NR	30	42	2 SER 1 IOD	0	Artix-7
**Arpi-10 [7]**	321.5	NR	0.3/0.65	NR	NR	4 SER 4 IOD	0	Virtex-4
**Imre-10 [6]**	312	NR	0.6/NR	NR	NR	4 SER 4 IOD	0	Virtex-4
**Fino-24 [74]**	100	42	0.35/NR	NR	NR	4 SER 3 IOD	0	Artix-7
			**Two-stage**					
**Dong-20 [75]**	78.13	35	0.8/0.94	199	347	1 SER 1 IOD	0	Kintex-7
**Wang-24 [77]**	10.05	19.81	2.85/13.61	293	385	0	0	Virtex-7
**Wang-24 [77]**	4.57	22.88	4.36/18.26	440	570	0	0	Ultrascale
**Real-24 [76]**	415.84	186	0.2/0.15	102	115	1 IOD	0	Artix-7

**Table 2:** Low-resource TDC architectures: advantages and drawbacks.

Type	Advantages	Drawbacks
TDL	High-resolution	Calibration
		PVT
		Resource consumption
TDL:IODELAY	Resource-efficient	Moderate resolution
	PVT-compensated	Limited number
		Delay granularity
VRO	High-resolution	Resource consumption
		slightly linear
		high dead-time
GCO	Resource-efficient	Calibration
		PVT
MPSC	Resource-efficient	Moderate resolution
	Highly linear	
MPSC:SerDes	Resource-efficient	Moderate resolution
	Highly linear	Limited number

## Data Availability

Dataset available on request from the authors.
